# The Impact of a Heart Failure Educational Program for Physicians Varies Based Upon Physician Specialty

**DOI:** 10.14740/jocmr1790w

**Published:** 2014-03-31

**Authors:** Linda G. Park, Denis Mahar, Richard E. Shaw, Kathleen Dracup

**Affiliations:** a San Francisco VA Medical Center, 4150 Clement Street 181G, San Francisco, CA 94121, USA; b Contra Costa Regional Medical Center, 2500 Alhambra Ave, Martinez, CA 94553, USA; c California Pacific Medical Center, 2200 Webster St, San Francisco, CA 94115, USA; d Department of Physiological Nursing, University of California, 2 Koret Way, N611, San Francisco, CA 94143, USA

**Keywords:** Heart failure, Beta blocker, Primary care physician, Internal medicine, Family medicine, Practice variation, Education

## Abstract

**Background:**

Beta blocker (BB) doses are often suboptimal in heart failure (HF) management. Differences in BB management patterns may exist between physicians in family medicine (FM) and internal medicine (IM). The aims of this study were to compare: 1) BB doses and prescription patterns; and 2) health care utilization rates in patients cared for by all primary care physicians compared to an historical control group after an educational program on HF management. A subgroup analysis was performed between patients cared for by FM and IM physicians. A secondary aim was to assess physician knowledge scores and satisfaction.

**Methods:**

A historically controlled study was conducted among low-income, underserved HF patients (mean age 54.1 ± 13.1, males 70%, mean ejection fraction 28.2 ± 9.8%). Statistical methods included linear mixed models and Fisher’s exact tests to assess prescription patterns of BB dosing and health care utilization rates (all cause and HF related hospitalizations, emergency department use and clinic visits).

**Results:**

Among 135 patients (experimental N = 81 and control N = 54), a linear mixed model test of group by time interaction showed no difference in BB dosage (t = -0.12, P = 0.91). FM physicians prescribed significant changes in BB doses compared to IM physicians (P = 0.04), had higher numbers of clinic visits (P = 0.03) and reported greater satisfaction with the program.

**Conclusions:**

There was no difference in BB titration rates following an HF training intervention for physicians compared to historical controls. However, FM physicians had a greater change in prescribing practices compared to IM physicians. Educational programs targeting FM physicians may benefit HF patients and could potentially lead to greater adherence to clinical guidelines related to BB use and address gaps in providing HF care.

## Introduction

Reinforcement of evidence-based guidelines of heart failure (HF) remains an opportunity for continued professional development among practitioners who manage HF. HF is a complex clinical syndrome that requires a multifaceted therapeutic regimen and is associated with frequent hospitalizations, poor quality of life, high morbidity and 1-year mortality rates of up to 30% [1, 2]. Hospitalization and mortality rates vary substantially among geographic locations, representing marked differences in outcomes that are not explained by insurance status [2]. HF is the most common hospital discharge diagnosis among Medicare beneficiaries and is considered one of the most expensive conditions in the healthcare system due to frequent readmissions [3].

Among the availability of many therapeutic options for HF, the guidelines are clear about the recommendation for use of evidence-based medications such as beta blockers (BBs) to reduce morbidity and mortality in HF [1, 4]. Since the formal recommendation for BB use in the late 1990s, there was a slow uptake of prescribing appropriate dosage by practitioners, which was partly attributed to the previous contraindication of BB as HF therapy [5]. Over time, there has been a significant increase in BB use in the HF population, largely credited to campaigns and interventions stressing the practice statements of BB use and performance measures at hospitals with strict criteria for documented BB prescription [5-8].

BB doses are often prescribed at suboptimal levels [5, 8], which may not provide maximum benefit for HF patients. Reasons for inability to achieve maximum dosage may be related to adverse clinical response (for example, bradycardia, hypotension), adverse clinical symptoms (for example, fatigue) and exacerbation of comorbid conditions (for example, bronchospasm). However, other differences in BB management patterns may exist between physician groups due to differences in specialty interest and training. Studies have shown that HF patients treated by cardiologists have better clinical outcomes including adherence to treatments as well as reduced mortality and rehospitalization rates compared to HF patients who are treated by non-cardiologists [9-12].

HF patients are often treated by primary care physicians (PCPs), including physicians with specialties in family medicine (FM) and internal medicine (IM). There are limited data available about the impact of specialized HF training for PCPs and limited understanding about the differences in practice patterns of HF management between FM and IM physicians. Researchers who studied the predictors of increased prescription of BB by PCPs found physicians who were more confident about their knowledge of appropriate HF management had higher BB prescription rates [13]. Further research was recommended to study the impact of physician training and education on prescription patterns [13].

Therefore, we conducted a pilot study termed Specialty Training and Resources for Improved Outcomes and Adherence to National Guidelines in Congestive Heart Failure (STRONG CHF) directed at PCPs. The aims of this study were to examine the impact of a 1-day educational program by comparing: 1) BB doses and prescription patterns between HF patients who were treated by PCPs before and after the program; and 2) health care utilization rates with hospitalizations, emergency department (ED) use and clinic visits between groups. A subgroup analysis of BB doses, BB prescription patterns and health care utilization between FM and IM patients before and after the educational program was performed. A secondary aim was to assess PCP knowledge scores and satisfaction with the program.

## Methods

### Setting and participants

A historically controlled study [14] was conducted at a community, county hospital system in Northern California servicing a multiethnic population of low income, uninsured, underserved HF patients. This county hospital system had a limited number of specialty physicians and did not employ any cardiologists at the time of the program. The study was approved by the Institutional Review Board at Contra Costa Regional Medical Center. Written informed consents were not deemed necessary as the protocol required data acquisition from an established database of electronic and paper medical records without patient contact.

The intervention was considered a pilot study to determine feasibility and acceptability, thus a power analysis and sample size calculation were not conducted *a priori*. All subjects in this sample were abstracted from a database of patients who were seen in one of six community-based clinics by the participating PCPs for a diagnosis of HF or cardiomyopathy. Data on BB doses and prescription practices were collected over a period of 14 months after the educational program for the experimental group (December 2009 to January 2011). The same data were collected for the control group who were seen by the trained PCPs for up to 23 months before the educational program (January 2008 to November 2009).

Patients were included for analysis if they had two or more clinic visits within 6 months with one of the trained PCPs, had left ventricular systolic dysfunction with an ejection fraction of ≤ 45% and were prescribed an evidence-based BB (or metoprolol tartrate). Although immediate release metoprolol tartrate is not considered an evidence-based BB for HF therapy [4], it was included in the analysis as it was often prescribed in lieu of sustained release metoprolol succinate because the latter was more costly in the hospital system. Patients who were previously taking BBs or had newly prescribed BBs were included in the analysis. Patients who were taking other BBs such as propanolol or atenolol were excluded.

### Intervention

Twelve participating PCPs, including five FM and seven IM physicians, who were all employed by the county health system, participated in the same educational program. During the 6-h educational training session, HF specialists instructed PCPs on the fundamentals of HF management and used case-based studies that emphasized appropriate BB dosing based on current guideline recommendations.

Health systems changes were implemented to support the STRONG CHF project in following HF patients in a more timely manner by the trained providers. One dedicated HF visit slot was reserved in each PCP’s continuity clinic. A standard clinic note template was created for HF patients who were seen by these providers. Follow-up visits focused on HF symptom management plus timely and efficient medication dosing. Patients in this study continued to receive primary care services from their established PCP.

### Outcomes

BB doses were compared between patients before and after physicians participated in the educational program in order to answer the major aim of the study. In addition, BB prescription patterns were compared in the following six categories: 1) dose unchanged, 2) up-titrated to maximum dose, 3) up-titrated but did not reach maximum dose, 4) already at maximum dose, 5) was at maximum dose then decreased, and 6) was not at maximum dose then decreased dose. Equivalency doses were established across different BBs, which allowed direct comparison. Data on a subgroup analysis detecting differences in BB doses and prescribing patterns between specialties were obtained.

To answer the next major aim, health care utilization rates were gathered on the number of all cause and HF related hospitalizations, ED use and clinic visits. Data on health care utilization were generally limited to services within the county health system unless physician records reported use at other medical institutions. A subgroup analysis on health care utilization was also performed between specialties.

For the secondary aim, surveys were administered to the participating PCPs to assess knowledge before and after the educational program. A survey about PCP satisfaction with the program was sent after the prospective study data were collected.

### Statistical analysis

Descriptive and clinical variables were compared between groups using t tests for continuous variables and Fisher’s exact tests for categorical variables. A linear mixed model examined differences in BB dose between the experimental and control groups and was employed to detect change over time with consideration to missing data using SPSS 18.0 (Chicago, IL). The basic design has one between participants (fixed) factor, group, with two levels (experimental and control) and measures of BB dose change for up to seven clinic visits. Differences in BB prescription patterns were compared using Fisher’s exact test analyses. Similar statistical applications for BB management were performed between FM and IM physicians for the specialty subgroup analysis. Fisher’s exact tests were used to detect differences in health care utilization rates between the study groups.

## Results

### Study participants

**Figure 1:**
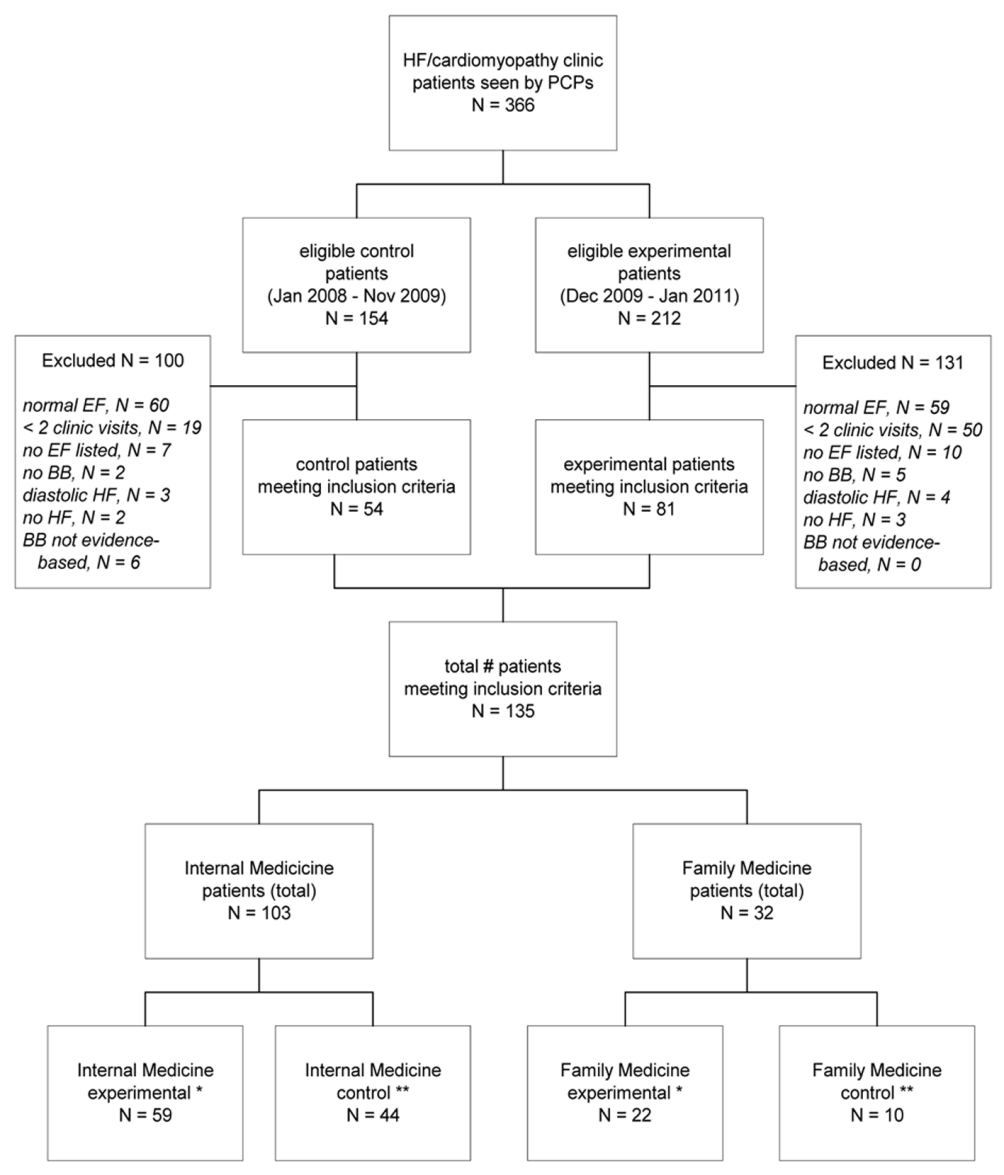
Groups identified and analyzed. BB: beta blocker; CV: clinic visits; FM: family medicine; HF: heart failure; IM: internal medicine; PCP: primary care physician. *Subgroup analysis compared IM and FM experimental groups. **Subgroup analysis compared IM and FM control groups.

The selection of study participants and groups compared are visually represented in Fig. 1. Medical records of historical control patients who received care from the participating PCPs 2 years prior to the intervention (N = 54) were compared to patients cared for following the educational program (N = 81). No sociodemographic or clinical differences were noted between experimental and control patients treated before or after the program (P > 0.05) (Table 1). The mean age of this sample was 54.1 ± 13.1 years, which is younger than typical HF patients because of the high incidence of illicit drug use among this group of patients (39%). Other key participant characteristics included a mean ejection fraction of 28.2 ± 9.8 and mean serum creatinine of 1.5 ± 1.0.

**Table 1:** Patient Characteristics of Experimental and Control Groups

	Experimental (N = 81)	Control (N = 54)	P value
Age (years ± SD)	54.1 ± 12.1	54.1 ± 14.5	0.98
Gender			0.54
Male	58 (71.6)	36 (66.7)	
Female	23 (28.4)	18 (33.3)	
HF etiology			0.40
Dilated	62 (76.5)	38 (70.4)	
Ischemic	16 (19.8)	14 (25.9)	
Not found	3 (3.7)	2 (3.7)	
Illicit drug use			0.77
Yes	32 (39.5)	20 (37.0)	
No	49 (60.5)	34 (63.0)	
LVEF			0.72
< 30%	52 (64.2)	33 (61.1)	
≥ 30%	29 (35.8)	21 (38.9)	
Serum creatinine			0.70
< 1.2 mg/dL	40 (49.4)	28 (52.8)	
≥ 1.2 mg/dL	41 (50.6)	25 (47.2)	
Mean serum creatinine (mg/dL ± SD)	1.3 ± 0.46	1.7 ± 1.4	0.05
β-type natriuretic peptide			0.73
< 100 pg/mL	6 (7.4)	3 (5.6)	
≥ 100 pg/mL	61 (75.3)	39 (72.2)	
Not found	14 (17.3)	12 (22.2)	
Mean BNP (pg/mL ± SD)	491.1 ± 1,037.4	329.3 ± 903.8	0.35

Abbreviations: BNP: β-type natriuretic peptide; HF: heart failure; LVEF: left ventricular ejection fraction; SD: standard deviation. Number of patients (%) reported unless otherwise specified.

In a subgroup analysis between FM and IM physicians, HF patients were more frequently followed by IM physicians (N = 103) compared to FM physicians (N = 32). Age was significantly higher in patients of IM physicians with a mean of 55.6 ± 13.6 years compared to 49.3 ± 9.8 years for patients of FM physicians (P = 0.02) (Table 2). Although differences in BNP and creatinine did not achieve significance, a trend was noted showing IM physicians followed older patients who potentially had more comorbidities.

**Table 2:** Patient Characteristics of Family Medicine and Internal Medicine Physicians

	Family medicine (N = 32)	Internal medicine (N = 103)	P value
Age (years ± SD)	49.3 ± 9.8	55.6 ± 13.6	0.02*
Gender			
Male	23 (71.9)	71 (68.9)	0.75
Female	9 (28.1)	32 (31.1)	
HF etiology			
Non-ischemic	26 (81.3)	75 (72.8)	0.59
Ischemic	5 (15.6)	25 (24.3)	
Illicit drug use			
Yes	11 (34.4)	41 (39.8)	0.58
No	21 (65.6)	62 (60.2)	
Mean LVEF (% ± SD)	28.16 (10.1)	28.24 (9.7)	0.97
Mean serum creatinine (mg/dL ± SD)	1.2 (0.4)	1.6 (1.1)	0.10
Mean BNP (pg/mL ± SD)	618 (345.9)	787.3 (871.9)	0.36

Abbreviations: BNP: β-type natriuretic peptide; HF: heart failure; LVEF: left ventricular ejection fraction; SD: standard deviation. Number of patients (%) reported unless otherwise specified. *P < 0.05.

Furthermore, patients who were treated by PCPs before and after the program in each of the specialties were analyzed. There were 59 experimental and 44 control patients in the IM group, while there were 22 experimental and 10 control patients in the FM group.

### Outcome: BB dose change and prescription patterns between experimental and control groups

The three BBs that were prescribed by PCPs during this study were carvedilol, metoprolol succinate and metoprolol tartrate. The majority of patients in this sample were prescribed carvedilol (72.8% in experimental, 53.7% in control). No significant difference in the choice of BB medication prescribed was found between the experimental and control groups (P = 0.73).

When examining BB doses over time, the linear mixed model test of the group by time interaction showed no difference between the experimental and control groups in linear change trajectories for BB dose change (t = -0.12, 95% CI: 8.11 - 7.18, P = 0.91). The mean BB dose for experimental patients was 143.3 ± 103.4 mg, whereas the mean dose for control patients was 141.4 ± 101.7 (maximum of 200 mg).

An analysis of BB prescription patterns revealed that of the 135 patients in the study, 40.7% of the experimental group compared to 51.8% of the control group had their BB dose up-titrated to higher or maximum doses. There were no significant differences between the proportion of experimental and control patients who did not have BB doses changed (P = 1.00), had BB up-titrated to maximum dose (P = 0.15), had BB up-titrated but not to maximum dose (P = 1.00), or were already at maximum dose (P = 0.24) (Table 3). Furthermore, there were no differences between groups when BB doses were decreased from a maximum dose of 200 mg or less.

**Table 3:** Beta Blocker Prescription Patterns: Experimental and Control Groups

	Experimental (N = 81)	Control (N = 54)	P value
No dose change	20 (24.7)	13 (24.1)	1.00
Up-titrated to max dose	15 (18.5)	16 (29.6)	0.15
Up-titrated but not to max dose	18 (22.2)	12 (22.2)	1.00
Already at max dose	23 (28.4)	10 (18.5)	0.24
Max dose then decreased	3 (3.7)	3 (5.6)	0.68
Not at max dose then decreased	2 (2.5)	0	0.08

Number of patients (%) reported.

### Outcome: health care utilization between experimental and control groups

Health care utilization over 6 months was examined between experimental and control groups in terms of all cause vs. HF related hospitalizations, ED use and clinic visits. Patients were seen for HF related clinic from two to seven times with a mean of 3.8 and 3.2 clinic visits for experimental and control groups, respectively (P = 0.06). Overall, there were no other differences between groups in health care utilization (Table 4).

**Table 4:** Health Care Utilization: Experimental and Control Groups

	Experimental (N = 81)	Control (N = 54)	P value
All-cause hospitalization	0.32 ± 0.83	0.44 ± 0.90	0.42
HF hospitalizations	0.16 ± 0.54	0.30 ± 0.69	0.20
All-cause ED visits	0.81 ± 1.26	0.70 ± 1.19	0.61
HF ED visits	0.37 ± 1.16	0.31 ± 0.72	0.75
Total clinic visits	6.83 ± 3.50	6.50 ± 3.61	0.60
HF clinic visits	3.78 ± 1.71	3.24 ± 1.39	0.06

Mean number of visits ± SD reported.

### Outcome: subgroup analysis between specialties on BB dose change and prescription patterns

A subgroup analysis was conducted to determine if there were any differences in BB dose change and prescription patterns according to physician specialty (FM compared to IM patients). A linear mixed models analysis showed there were significant differences between FM and IM physicians in linear change trajectories for BB dose change (t = 2.13, 95% CI: 4.28 - 172.72, P = 0.04) with more BB dose change reflected in the FM patients.

The BB total dose change across clinic visits for FM and IM physicians was significantly different. Mean doses of BB change were 70 mg for control patients (N = 10) and 160 mg for experimental patients (N = 22) among FM physicians. In contrast, mean doses of BB change were 147 mg for control patients (N = 44) and 120 mg for experimental patients (N = 59) for IM physicians. It is unclear why BB doses decreased for IM patients but it is hypothesized that HF patients became more debilitated and were not able to tolerate higher doses as time progressed.

In examining BB prescription patterns, among the patients of FM physicians, 31.8% compared to 44% of the patients of IM physicians had their BB dose up-titrated to higher or maximum doses as a result of the educational program. No differences were found between FM and IM patients when comparing BB prescription patterns (Table 5).

**Table 5:** Beta Blocker Prescription Patterns: Specialty Groups

	Internal medicine	Family medicine	P value
Experimental	(N = 59)	(N = 22)	
No dose change	18 (30.5)	2 (9.1)	0.08
Up-titrated to max dose	12 (20.3)	3 (13.6)	0.75
Up-titrated but not to max dose	14 (23.7)	4 (18.2)	0.77
Already at max dose	14 (23.7)	9 (40.9)	0.17
Max dose then decreased	1 (1.7)	2 (9.1)	0.18
Not at max dose then decreased	0	2 (9.1)	0.07
Control	(N = 44)	(N = 10)	
No dose change	11 (25)	2 (20)	1.00
Up-titrated to max dose	13 (29.5)	3 (30)	1.00
Up-titrated but not to max dose	9 (20.5)	3 (30)	0.67
Already at max dose	9 (20.5)	1 (10)	0.67
Max dose then decreased	2 (4.5)	1 (10)	0.47
Not at max dose then decreased	0	0	-

Number of patients (%) reported.

### Outcome: subgroup analysis between specialties on health care utilization

The same analysis for health care utilization (hospitalizations, ED visits, clinic visits for all cause and HF related) was performed among experimental vs. control groups in each specialty group. The subgroup analysis between specialties showed FM experimental patients had more HF related clinic visits than IM experimental patients over 6 months after the educational program (4.45 ± 2.02 and 3.53 ± 1.52 clinic visits, respectively, P = 0.03) (Table 6). No other differences in all cause or HF related hospitalizations and ED visits were found.

**Table 6:** Health Care Utilization: Specialty Groups

	Internal medicine	Family medicine	P value
Experimental	(N = 59)	(N = 22)	
All-cause hospitalization	0.37 ± 0.93	0.18 ± 0.50	0.36
HF hospitalizations	0.20 ± 0.61	0.05 ± 0.21	0.24
All-cause ED visits	0.92 ± 1.28	0.55 ± 1.18	0.24
HF ED visits	0.46 ± 1.32	0.14 ± 0.47	0.27
Total clinic visits	7.05 ± 3.70	6.23 ± 2.89	0.35
HF clinic visits	3.53 ± 1.52	4.45 ± 2.02	0.03*
Control	(N = 44)	(N = 10)	
All-cause hospitalization	0.50 ± 0.98	0.20 ± 0.42	0.35
HF hospitalizations	0.32 ± 0.74	0.20 ± 0.42	0.63
All-cause ED visits	0.75 ± 1.18	0.50 ± 1.27	0.55
HF ED visits	0.36 ± 0.78	0.10 ± 0.32	0.30
Total clinic visits	6.36 ± 3.65	7.10 ± 3.51	0.57
HF clinic visits	3.18 ± 1.35	3.50 ± 1.58	0.52

Mean number of visits ± SD reported. *P < 0.05.

### Outcome: physician knowledge and satisfaction scores

An HF knowledge test was administered before and immediately following the educational program. Significant improvement in HF knowledge scores among all PCPs were found with correct pretest scores of 68% and posttest scores of 93%.

**Figure 2:**
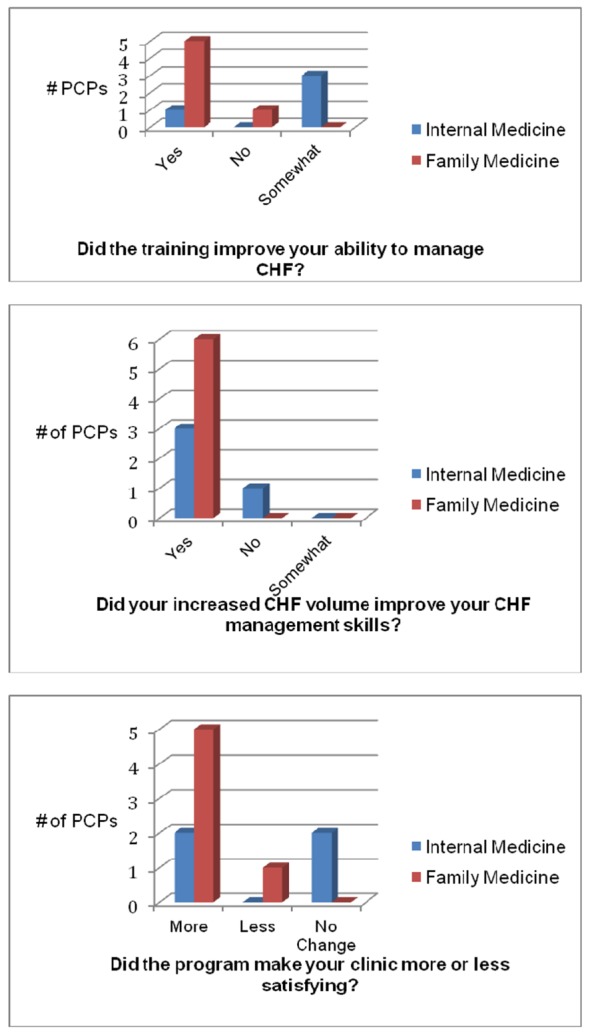
Satisfaction survey (internal medicine N = 4, family medicine N = 6). PCP: primary care physician.

Nine out of 12 PCPs completed the follow-up satisfaction questionnaire (Fig. 2). In comparison to IM physicians, FM physicians reported improved ability to manage HF, improved management skills due to an increased number of HF patients and more satisfying clinics as a result of the STRONG CHF program.

## Discussion

The HF educational program did not result in significantly higher BB dose change or improved BB prescription patterns by PCPs. BB doses were suboptimal for HF patients, although the clinical reasons for restricting maximal dosage were not explored in this study. Importantly, a subgroup analysis showed the educational program on HF topics was shown to benefit HF patients in achieving significant BB dose change in FM physicians compared to IM physicians. However, a comparison between specific BB prescription patterns (for example, up-titration to maximum dose) between patients cared for before and after the educational program did not reveal any differences.

Health care utilization between the experimental and control groups was not significantly different, although there was a trend that HF patients had more clinic visits following the educational program. The subgroup analysis between specialties showed FM patients had more clinic visits than IM patients after the educational program, which may coincide with the significant finding of BB dose change in the FM group.

Lastly, FM physicians reported more satisfaction and practice improvement from the educational program compared to the IM physicians. The reports of higher satisfaction may be important information to tailor future programs in this cohort of physicians.

Several limitations may apply in this pilot study. The small sample size may not have allowed for significant differences to be noted between the experimental and control groups. The results of this younger patient population with socioeconomic challenges and a higher incidence of illicit drug use may be significantly different from other HF populations who are older, more attentive to their health care needs and have better access to health care providers. Thus, the results may be difficult to generalize. The patient characteristics between FM and IM groups showed IM patients were older, which may have led to bias, although other clinical factors were not significantly different. In addition, due to the high demand of clinic appointments with the PCPs involved in this study, there may have been a significant delay in scheduling follow-up visits, which in turn caused a delay in time and dosage of BB titration. The medical and socioeconomic complexities of patients in this study may have attenuated the effects of the educational program. In the subgroup analysis, characteristics of PCPs were not explored *a priori* (for example, age, years of practice). The IM physicians may have been highly experienced in managing complex HF patients given the specialty limited care that was available in this county hospital setting; therefore, the educational program may not have been as salient for them as for other physician groups.

The impact of future educational programs on specialty topics such as HF management for PCPs should be examined further, particularly in resource limited settings. Given the growing population of older adults that will be treated for HF, specialized educational training of PCPs appears to be a simple, feasible and practical solution to offering HF services, particularly in resource limited areas. Trained FM or IM physicians could potentially fill gaps in HF care in resource limited settings such as public health care systems or rural areas. Future research can replicate the educational program in health care systems that have few cardiologists. Specialty care for uninsured and underinsured patients in the United States is in short supply [15]; therefore, health care institutions require efficient and sustainable systems founded upon well-trained PCPs. A unique and feasible practice model in which FM and IM physicians expand their scope of work to provide specialty care in HF clinics with an interdisciplinary team is necessary to meet the growing population of HF patients. This model would allow for cardiologists to serve as consultants for very complicated or advanced HF patients and would allow them time to manage other complex cardiac patients.

Previous research has shown BB doses are often suboptimal, but are higher in HF patients who are managed by a cardiology specialist or specialized HF disease management programs [16-18]. Future research comparing PCPs after a specialized educational program for BB management in HF patients with cardiologists or other specialized HF disease management programs will determine whether an educational program can achieve comparable outcomes.
